# Enhanced chemical and physical defense traits in a rice cultivar showing resistance to leaffolder infestation

**DOI:** 10.1007/s44297-023-00010-z

**Published:** 2023-10-23

**Authors:** Wenyi Zhao, Yunqi Zhuang, Yumeng Chen, Yonggen Lou, Ran Li

**Affiliations:** Key Laboratory of Biology of Crop Pathogens and Insects of Zhejiang Province, Institute of Insect Sciences, Zhejiang University, Hangzhou, 310058 China

**Keywords:** Leaffolder, Rice resistance, Salicylic acid, Flavonoids, Silicified trichomes

## Abstract

The leaffolder (LF), *Cnaphalocrocis medinalis*, poses a significant threat to rice crops. The larvae feed on rice leaves, damaging both the epidermal and mesophyll tissues, and engage in leaf-folding behaviors to build shelters that can lead to significant yield losses. In this study, we discovered a rice cultivar, P213, that exhibits resistance to LF infestation. When compared to the susceptible cultivar XiuShui11 (XS11), the larvae on P213 exhibited reduced weight and took longer to build their silk-leaf shelters. Through a side-by-side analysis of phytohormones, metabolites and transcriptomes in the two cultivars, we found that jasmonate (JA)-induced defenses are not the main contributors to P213 resistance against LF. Rather, salicylic acid and flavonoids emerge as potential chemical defenses against this insect pest. Furthermore, the resistant cultivar P213 exhibited a greater density of micro hairs and long macro hairs, the latter of which were absent in the susceptible cultivar. The silicon contents in leaf tissues were also more prominent in the resistant cultivar, suggesting that the physical defenses against LF are enhanced in P213. These findings shed new light on the mechanisms of rice resistance to LF and provide a foundation for future research into the genetic basis of this resistance.

**Supplementary Information:**

The online version contains supplementary material available at 10.1007/s44297-023-00010-z.

## Introduction

The leaffolder (LF), *Cnaphalocrocis medinalis* Guenée (Lepidoptera: Pyralidae), is a major pest in the rice-growing regions of Asia [1]. Significant yield losses can occur if attacks of this insect occur during the middle or late stages of rice development [2]. The larvae produce silk to fold the leaves longitudinally and consume the upper epidermis as well as mesophyll tissues. This specific feeding pattern gives rise to linear, pale white stripes on the leaves [1], which significantly impair photosynthetic activity, leading to yield losses—especially when the flag leaves are damaged during the booting stage [3]. The adoption of novel agricultural practices in paddy fields—including the cultivation of high-yielding semidwarf varieties, the implementation of irrigation systems, and the use of high levels of nitrogenous fertilizers and pesticides—has led to an increased incidence of LF across various Asian countries. In China alone, LFs affect an average annual area exceeding 20 million hectares, resulting in rice yield losses of up to 0.76 million metric tons [4]. Insecticidal control has traditionally been used to manage LF populations. However, long-term application of pesticides comes with negative effects such as environmental pollution, the development of insecticide resistance, and the disruption of existing biological control mechanisms. Harnessing genetic diversity to improve crop resistance has been proven to be a more sustainable, economical, and environmentally safe way to protect crops against insect pests.

Plants have a diverse array of chemical and physical defense mechanisms to cope with herbivores [5]. Chemical defenses include the biosynthesis of specialized metabolites and defensive proteins. Plants produce a plethora of specialized metabolites throughout their lifespan, many of which serve anti-herbivore functions by acting as toxic or anti-feedant compounds [6]. For instance, when plants of the Brassicaceae family are damaged, sulfur- and nitrogen-containing compounds, known as glucosinolates, are hydrolyzed to produce isothiocyanates, which are toxic to many herbivores [7]. Similarly, nicotine, an alkaloid found in tobacco plants, is toxic to nonspecialist herbivores, as it binds to nicotinic acetylcholine receptors in neuromuscular junctions, impairing locomotor activity [8, 9]. Additionally, another type of plant defense compound, volatile organic compounds (VOCs), can have several functions: they can directly repel herbivores or act indirectly by attracting natural enemies of herbivores. For example, certain sesquiterpenes emitted by tomato plants have been shown to repel the whitefly *Bemisia tabaci* [10]. In the case of rice, VOCs such as linalool and caryophyllene attract parasitoid wasps that target the brown planthopper (BPH) [11]. More recently, a novel defensive function of some specialized plant metabolites involves their toxic effect on beneficial yeast-like endosymbionts that provide nutritional benefits to insects [12]. In addition, some plant-produced defense proteins (e.g., proteinase inhibitors, PIs) are known to reduce herbivore performance by inhibiting the activity of digestive enzymes [13]. Most plant chemical defenses are inducible, being either produced or increased in response to herbivore attack, and are regulated through phytohormone signaling [14]. Among all phytohormones, jasmonates (JAs) are the main regulators of plant inducible defenses against herbivores [5, 15]. Unlike chemical defenses, which are mostly inducible, many physical defenses are constitutive barriers against herbivores, such as spines, trichomes, and thick cuticles [16]. The function of these physical defenses ranges from acting as feeding and ovipositing deterrents to reducing plant palatability.

The brown planthopper (BPH) is a major pest of rice, causing significant yield losses. Rice resistance to BPH has been extensively studied. Many BPH resistance genes have been cloned, and BPH-elicited defense responses have been identified [15, 17–23]. These include chemical defenses, such as sakuranetin, p-coumaroyl putrescine, and linalool [11, 12, 24]. On the other hand, the study of rice defense against LF has received less attention. To date, no LF resistance gene has been cloned. Some rice responses to LF attack are known to be inducible and JA dependent, such as the accumulation of trypsin PIs (TrypPIs), peroxidase, polyphenol oxidase, and phenolamides [25–27]. As a result, silencing or creating knockout mutants of rice genes involved in JA biosynthesis or signaling leads to reduced resistance to LF infestations. Other studies have shown that plant PIs could act against LF by ectopically expressing the potato proteinase inhibitor II gene in rice [28]. Physical defenses have also been reported to play a role in the rice-LF interaction. The nonessential element silicon (Si) has been positively correlated with LF resistance by two means: as a direct physical defense [29] and indirectly by priming JA-mediated defense responses [26]. However, most of the defensive traits of rice against this important insect pest remain unknown.

In the present study, we address this question using a comparative analysis of rice chemical and physical defenses against LF using a sensitive and newly identified resistant cultivar. The resistant cultivar P213 was selected based on field observations, while the relatively susceptible cultivar Xiushui 11 (XS11) served as a control. A series of bioassays were conducted to evaluate the reduced performance of LF in the P213 cultivar. We then quantified phytohormone levels and evaluated both chemical and physical defense mechanisms in P213 and XS11, aiming to identify rice traits correlated with LF resistance. To further elucidate the underlying molecular pathways associated with these defensive traits, we conducted a comparative transcriptome analysis between P213 and XS11. Our findings suggest that the salicylic acid (SA) signaling pathway, along with flavonoids and silicified trichomes, likely contributes to LF resistance in the P213 cultivar.

## Results

### Reduced LF larval performance on the resistant rice cultivar P213

**Fig. 1:**
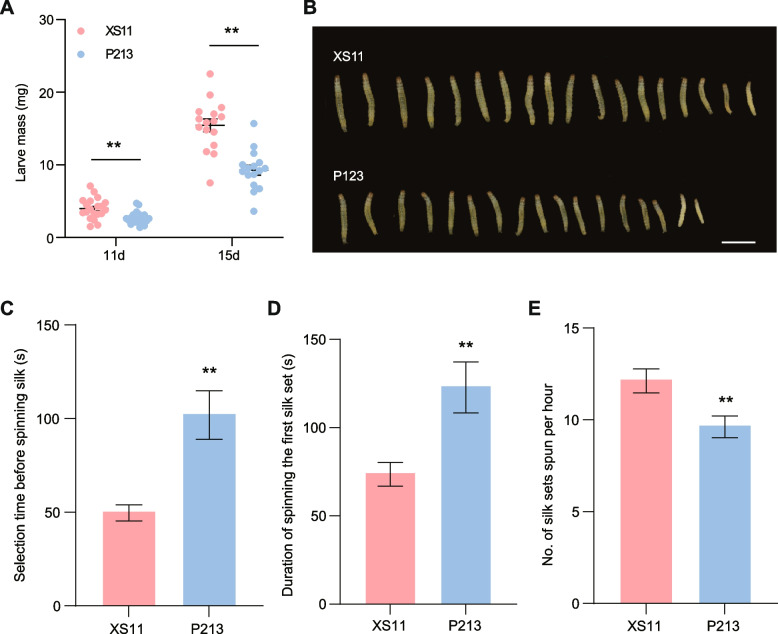
Leaffolder (LF) performance on XS11 and P213 plants. **A** Mean (± SE, *n* = 17-18) weight of LF larvae fed on XS11 and P213 plants. A single newly hatched LF larva was allowed to feed on each of the XS11 and P213 plants. The larval mass was recorded on days 11 and 15. **B** The picture depicts LF larvae. Bar = 1 cm. **C** Mean selection time (± SE, *n* = 14) of fourth-instar LF larvae on XS11 and P213 plants before spinning silk. **D** Mean duration time (± SE, *n* = 14) of a fourth-instar LF larva to spit the first set of silk on XS11 and P213 plants. **E** The mean number of silk sets (± SE, *n* = 14) spun per hour by a fourth-instar LF larva on XS11 and P213 plants. Asterisks indicate significant differences in P213 compared with XS11 plants (**, *P* < 0.01; Student’s *t* test)

In previous field trials, we identified an LF-resistant japonica cultivar, P213, whose genetic background is unknown. To evaluate how P213 plants affect LF performance, a series of bioassays with this cultivar and a more susceptible japonica cultivar, Xiushui 11 (XS11), was conducted. While the survival rates of newly hatched LF larvae did not differ between the two cultivars (Fig. 1A), larvae feeding on P213 plants gained significantly less weight when compared to XS11 plants, with a mass reduction of 33% and 40% on days 11 and 15, respectively (Fig. 1B). The amount of leaf area damaged by LF larvae in both cultivars was then quantified, and we did not find significant differences among cultivars (Figs. S1B and C). Leaf folding, a characteristic behavior of LF larvae for sheltering, was also examined. Fourth-instar larvae on P213 took a significantly longer time to begin spinning silk (Fig. 1C) and needed more time to spin the first set of silk (Fig. 1D). Consequently, the overall rate of silk-spinning per hour was reduced on P213 plants (Fig. 1E). These findings indicate that the cultivar P213 is relatively more resistant to LF infestation.

### Enhanced salicylic acid (SA) accumulation in the resistant rice cultivar P213

**Fig. 2:**
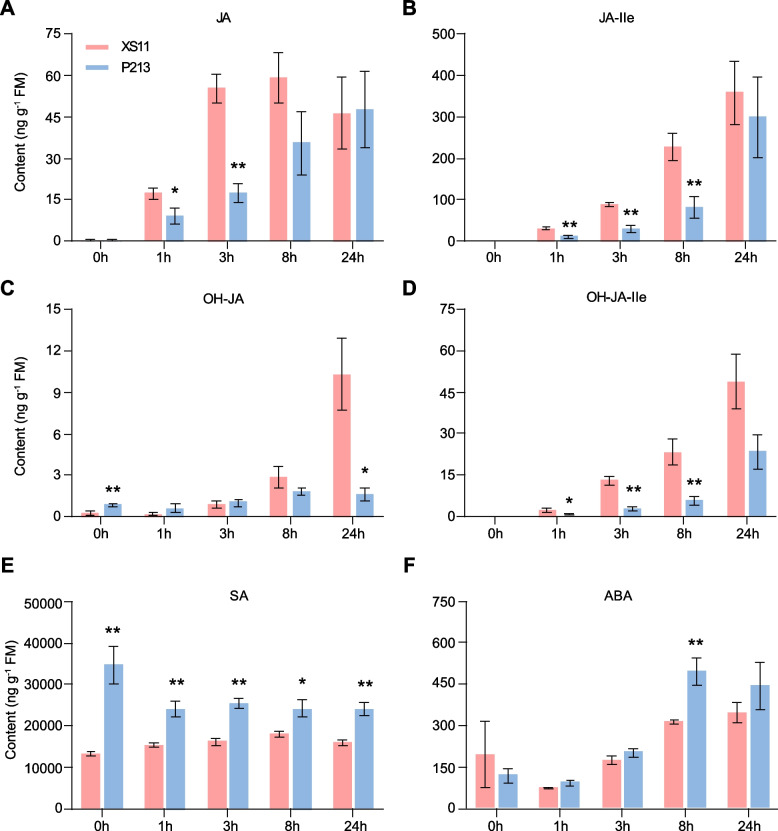
LF-induced phytohormone levels in the leaves of XS11 and P213 plants. Mean concentrations (± SE, *n* = 5) of JA **A**, JA-Ile **B**, 12-hydroxy-JA (OH-JA) **C**, 12-hydroxy-JA-Ile (OH-JA-Ile) **D**, salicylic acid (SA) **E** and abscisic acid (ABA) **F** in the leaves of XS11 and P213 plants. A third-instar LF larva was allowed to feed on the first fully expanded leaf per plant at the indicated time points. Asterisks indicate significant differences in P213 compared with XS11 plants (*, *P* < 0.05; **, *P* < 0.01; Student’s *t* test)

The JA signaling pathway positively regulates rice defense against LF [27]. To determine whether the increased resistance to LF in P213 plants is associated with jasmonates (JAs), the LF-induced levels of several phytohormones were measured. Consistent with previous results, the levels of JA, its derivative JA-Ile, and the hydroxylated forms 12-hydroxy-JA (OH-JA) and 12-hydroxy-JA-Ile (OH-JA-Ile) were significantly increased in XS11 plants in response to LF attack and continued to increase throughout the feeding period (Fig. 2A-D). However, the accumulations of these four JAs were significantly lower in P213 than in XS11. Conversely, both basal and LF-elicited SA levels were significantly elevated in P213 (Fig. 2E). Additionally, the levels of abscisic acid (ABA) were only higher in P213 after 8 h of LF treatment (Fig. 2F). These hormonal profiles suggest that SA signaling could be a contributing factor to the LF resistance observed in P213.

### Limited role of proteinase inhibitors and phenolamides in LF resistance in the P213 cultivar

**Fig. 3:**
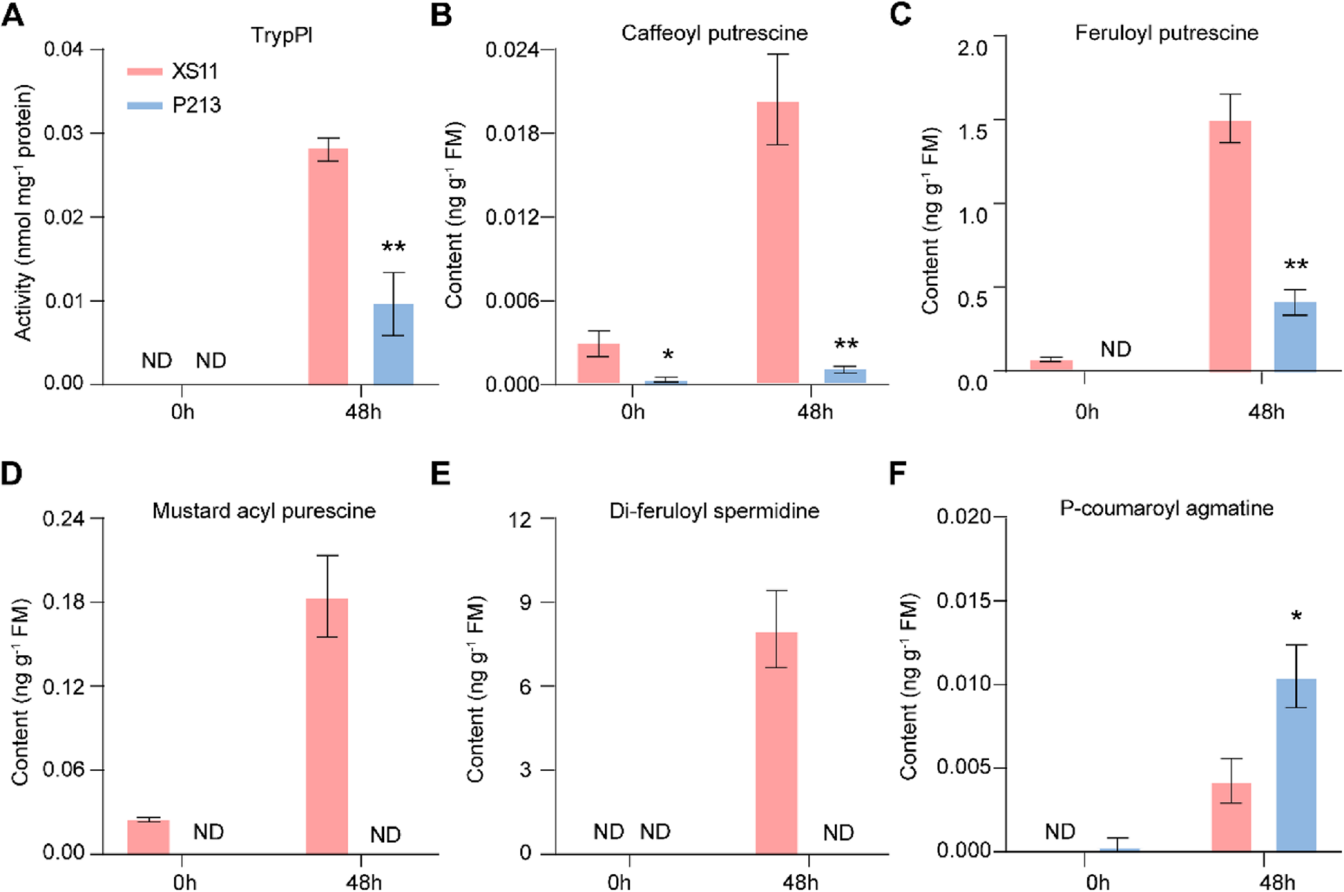
LF-induced trypsin proteinase inhibitor (TrypPI) and phenolamide levels in leaves of XS11 and P213 plants. **A** Mean TrypPI activity (± SE, *n* = 6) in P213 and WT plants under LF feeding. Mean concentrations (± SE, *n* = 7) of caffeoyl putrescine **B**, feruloyl putrescine **C**, mustard acyl putrescine **D**, di-feruloyl spermidine **E**, and p-coumarcoyl agmatine **F** in XS11 and P213 plants. A third-instar LF larva was allowed to feed on the first fully expanded leaf per plant at 0 h and 48 h. ND, not detected. Asterisks indicate significant differences in P213 compared with XS11 plants (*, *P* < 0.05; **, *P* < 0.01; Student’s *t* test)

Certain plant species produce proteinase inhibitors and phenolamides as defenses against noctuid larvae [28, 30, 31]. Herbivore infestation is known to induce the accumulation of these compounds in rice [15, 27]. To assess whether these chemical defenses are induced to higher levels in the P213 cultivar in response to LF attack, the contents of trypsin proteinase inhibitor (TrypPI) and five specific phenolamides were quantified. The basal levels of TrypPI in leaves were extremely low but increased significantly in response to LF feeding (Fig. 3A). Despite this, P213 plants displayed lower TrypPI levels than XS11 plants. Similarly, the concentrations of several phenolamides, including caffeoyl putrescine, feruloyl putrescine, mustard acyl putrescine, di-feruloyl spermidine and p-coumaroyl agmatine, were strongly upregulated in XS11 plants after LF feeding but remained relatively low in P213 (Fig. 3B-E). An exception was p-coumaroyl agmatine, which showed increased levels in P213 relative to XS11 (Fig. 3F). These results suggested that TrypPIs and phenolamides may not be key factors conferring LF resistance in the P213 cultivar.

### The P213 cultivar has elevated flavonoid contents

**Fig. 4:**
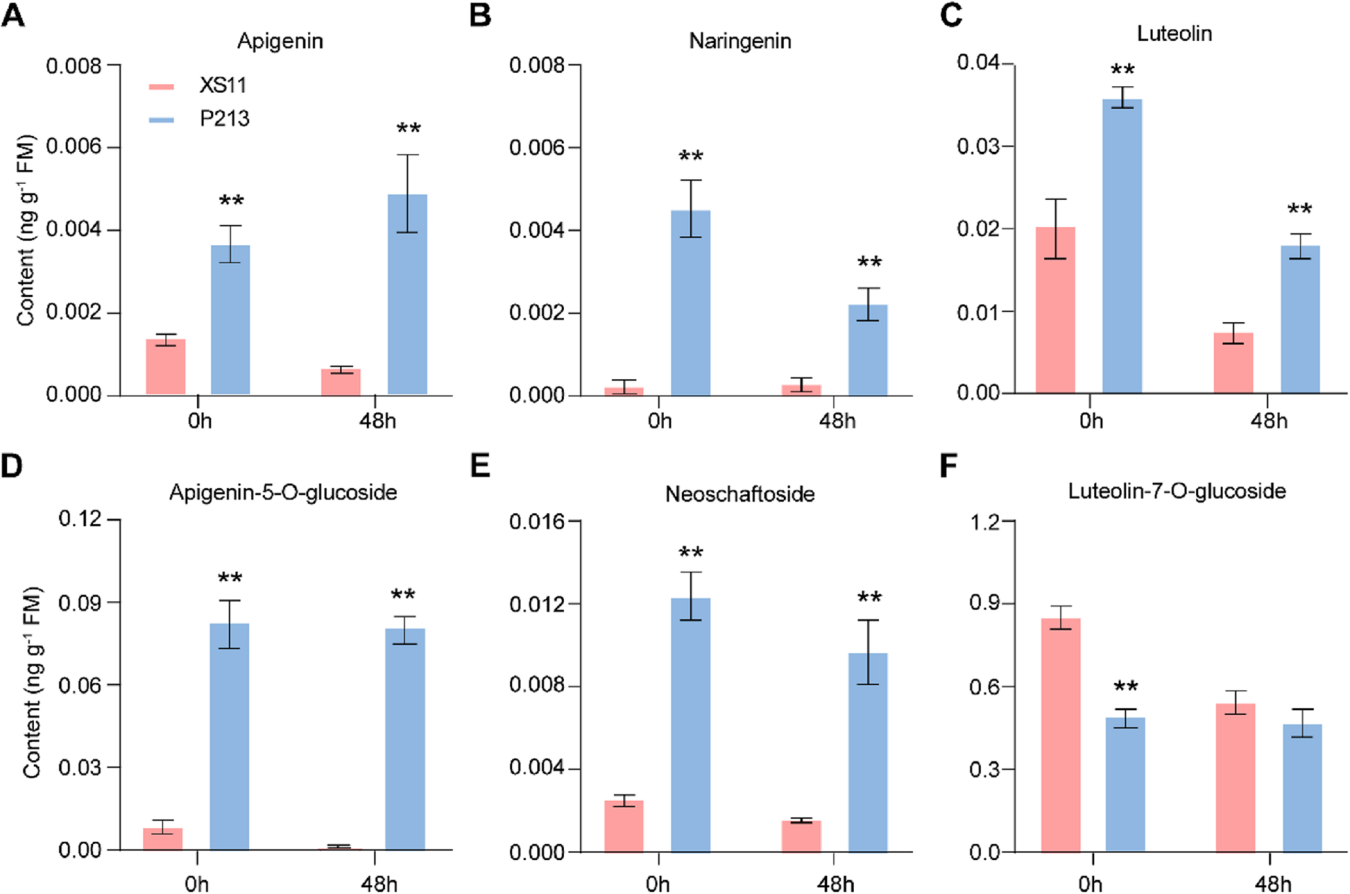
LF-induced flavonoid levels in the leaves of XS11 and P213 plants. Mean concentrations (± SE, *n* = 7) of apigenin **A**, naringenin **B**, luteolin **C**, apigenin 5-O-glucoside **D** and neoschaftoside **E** in XS11 and P213 plants. A third-instar LF larva was allowed to feed on the first fully expanded leaf per plant at 0 h and 48 h. Asterisks indicate significant differences in P213 compared with XS11 plants (**, *P* < 0.01; Student’s *t* test)

In a recent study, we discovered that the flavonoid sakuranetin serves as an anti-herbivore compound in rice [12]. To explore this further, the constitutive and LF-induced levels of several flavonoids were examined in the leaves of P213 and XS11 plants. Sakuranetin was not detected in LF-treated leaves, suggesting that LF feeding does not induce sakuranetin accumulation. In contrast to TrypPIs and phenolamides, the accumulation of most flavonoids decreased after LF treatment (Fig. 4A-F). On the other hand, the basal levels of apigenin, naringenin, luteolin, apigenin-5-O-glucoside, and neoschaftoside were significantly higher in P213 than in XS11. Conversely, the basal levels of luteolin-7-O-glucoside were higher in XS11 than in P213. Considering the potential anti-herbivore activities of flavonoids [32], their increased basal levels in P213 leaves may contribute to LF resistance.

### Silicified trichomes are more abundant in P213 plants

**Fig. 5:**
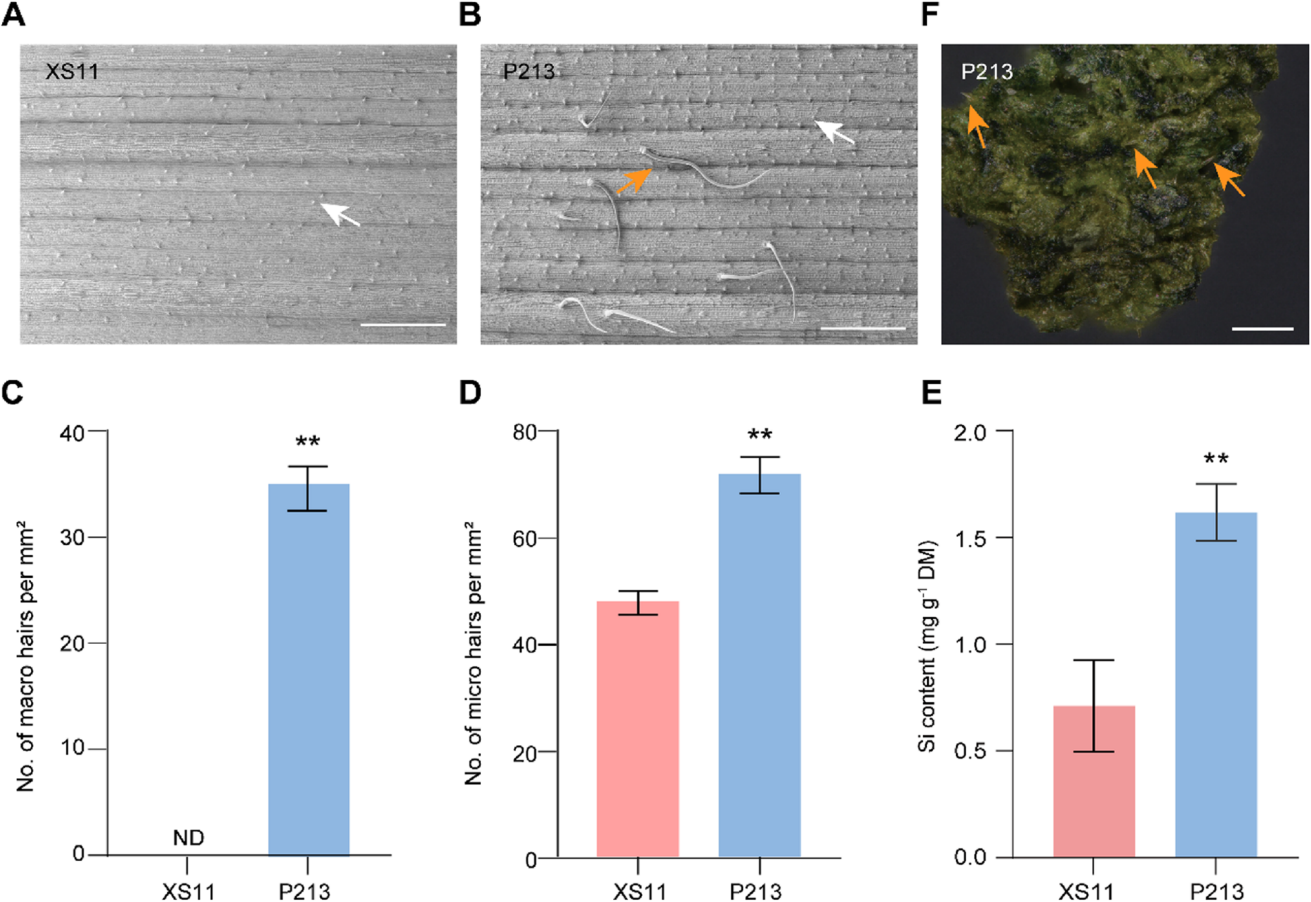
Comparison of silicified macro- and microhairs as physical defense features in the P213 and XS11 cultivars. Surface scanning electron microscopy (SEM) images of an XS11 **A** and a P213 leaf **B** Bar = 0.5 mm. The white arrow indicates micro hairs, and the orange arrow indicates macro hairs. Mean number (± SE, *n* = 4) of macro hairs **C** and micro hairs **D** on the surface of XS11 and P213 leaves. **E** Mean concentrations (± SE, *n*=11) of Si levels in the leaves of XS11 and P213 plants. ND, not detected. Asterisks indicate significant differences in P213 compared with XS11 plants. (**, *P* < 0.01; Student’s *t* test). DM, dry mass. **F** Undigested trichomes in the frass of LF larvae fed on P213 plants. The orange arrow indicates macro hairs. Bar = 100 μm

Beyond chemical defenses, physical barriers play an important role in plant defense against herbivores [5]. Our laboratory and field observations indicated an increased density of trichomes on the surface of leaves and leaf sheaths in P213 plants growing in these two conditions. Silicified trichomes are known to have a defensive role against chewing herbivores in rice [33, 34]. To quantify this, we examined the trichomes of P213 and XS11 leaves using scanning electron microscopy (SEM). Two primary types of trichomes, long macro hairs and micro hairs, were identified in these cultivars (Fig. 5A and B). Notably, long macro hairs were absent in XS11 leaves, whereas P213 plants exhibited a high density of this type of trichome (~35 per mm^2^) (Fig. 5C). In addition, the number of micro hairs was also significantly higher on P213 leaves than on the XS11 cultivar (Fig. 5D). Given that the types of trichomes are silicified, the Si contents were then measured in the leaves of P213 and XS11. In line with the SEM observations, the accumulation of Si was significantly higher in P213 than in XS11 (Fig. 5E). Furthermore, many undigested macro hairs were found in the frass of LF larvae when fed P213 leaves (Fig. 5F), suggesting that these silicified trichomes may reduce the digestibility of leaves by LF. In addition to trichomes, other physical defenses in P213 and XS11 were also evaluated, such as lignin and cellulose, which are components of the cell wall. Their levels were marginally lower in the leaves of P213 than in XS11 (Figs. S2A and B), suggesting that these two components are unlikely to contribute to the enhanced LF resistance in P213.

### Transcriptome analysis identifies key pathways for rice resistance to LF

**Fig. 6:**
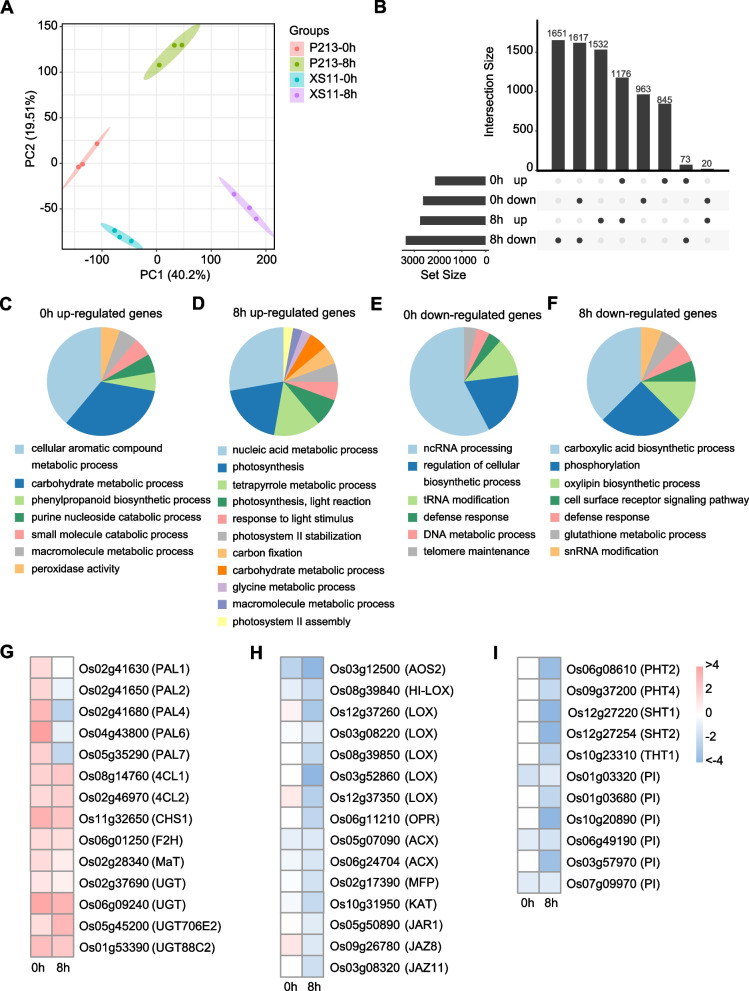
Transcriptional responses in leaves of P213 and XS11 in response to LF attack. **A** Principal component analysis (PCA) of RNA-seq data of control and LF-treated leaves of P213 and XS11 plants. **B** UpSet diagram showing the number of LF-induced differentially expressed genes (DEGs) in P213 compared with XS11 at 0 h and 8 h of treatment (left) and the top 8 interactions (bottom right) by size (top right). The cutoff of DEGs was fold-change > 2 and adjusted *p* value < 0.05. **C-F** Gene Ontology (GO) analysis of up- or downregulated DEGs in P213 compared with XS11 plants by CLUEGO. The percentage (%) terms (*P* > 0.05) per group and the representative GO term in each group are shown. The heatmap represents the transcription levels of phenylpropanoid biosynthetic genes **G**, JA biosynthetic and responsive genes **H**, and phenolamide and proteinase inhibitor (PI) biosynthetic genes **I** in P213 compared with XS11 plants. Numbers in the color key indicate log_2_(FC)

To investigate the molecular mechanism underpinning the differential resistance to LF in the P213 and XS11 cultivars, a comparative transcriptome analysis was conducted. Leaves of different plants were treated with LF larvae for 8 h, and nontreated leaves were used as controls. Gene expression was normalized using TPM (transcripts per million reads). Principal component analysis (PCA) revealed the clustering of samples within treatment groups, indicating the consistency of the treatments (Fig. 6A). This unbiased analysis also discriminated control and LF-treated samples from P213 and XS11 plants, highlighting global gene expression differences between cultivars. Subsequent analysis of differentially expressed genes (DEGs) revealed that 2,094 and 2,728 genes were upregulated in P213 compared with XS11 after 0 h and 8 h post-LF treatment, respectively, while 2,600 and 3,341 genes were downregulated (Fig. 6B left panel). The largest three sets were downregulated DEGs in LF-treated and untreated P213 and upregulated DEGs in LF-treated P213 (Fig. 6B right panel). Gene Ontology (GO) analysis of DEGs showed that upregulated genes in P213 were enriched in aromatic compound metabolism and phenylpropanoid biosynthesis (Fig. 6C). Specifically, five phenylalanine ammonia lyase (PAL) genes, which catalyze the initial steps of SA and phenylpropanoid biosynthesis, were expressed at higher levels in P213 than in XS11 under control conditions (Fig. 6G; Table S1). Several uridine diphosphate (UDP) glycosyltransferase (UGT) genes involved in flavonoid biosynthesis were upregulated in P213 in both control and LF-treated conditions. LF-induced upregulated genes in P213 were also enriched in photosynthetic processes (Fig. 6D), indicating a compensatory response to LF leaf damage. The increased tolerance to herbivory may enable P213 plants to better recover or proliferate post-LF infestation [35]. Conversely, downregulated genes in P213 were enriched in defense response and oxylipin biosynthetic process (Figs. 6E and F). These GO categories included genes associated with JA biosynthesis, JA response marker genes, and phenolamide and PI biosynthetic genes (Figs. 6H and I; Table S1). This result is consistent with the reduced JA levels observed in P213.

## Discussion

LF is a common insect pest in rice fields, and host resistance is considered a viable strategy to control LF, *C. medinalis.* Despite this, the molecular mechanisms and genetic basis of rice resistance to this pest remain largely unknown. Here, we aim to identify rice defensive traits against LF by comparing a resistant cultivar, P213, with a susceptible cultivar, XS11. Our assays indicate that P213 is resistant to LF; larval performance is impaired in P213 as they gained less weight when fed on these plants relative to XS11, although the overall feeding amounts were similar. LF elicits the accumulation of JA and JA-mediated defensive compounds such as phenolamides in XS11, a response that was lessened in the P213 cultivar. Conversely, SA and flavonoid levels were constitutively higher in P213. Transcriptome analysis of basal and LF-elicited leaves from different cultivars supports the above findings and indicates that SA and flavonoids may play a role in LF defense in P213 plants. In terms of physical defenses, LF larvae displayed a slower leaf-folding behavior in P213 relative to XS11, suggesting the existence of physical barriers on P213 leaves. Scanning electron microscopy (SEM) revealed a higher abundance of silicified trichomes, especially long macro hairs, on P213 leaves relative to the controls. Silicon content was also elevated in P213 leaves, further supporting its defensive role. Si has been broadly reported to be involved in LF resistance [26, 36, 37]. Given that LF larvae scrape cells of the upper epidermis and mesophyll tissues, the nonglandular silicified trichomes on the leaf surface serve as an effective physical defense against LF. A function that resembles that of rice against other chewing herbivores [33, 34].

Although JA plays an important role in LF resistance, direct measurements of JAs and JA-induced defense compounds indicated that JA signaling is not the main driver of rice resistance against LF in P213 plants. Salicylic acid is known to mediate plant resistance to pathogens as well as some insect herbivores [38]. Increasing evidence suggests a correlation between SA signaling and LF resistance. For instance, the exogenous application of SA was found to increase the accumulation of trypsin proteinase inhibitors (TrypPIs) in rice [39]. Furthermore, the application of methyl salicylate-induced changes in rice plants affected the growth and development of LF [40]. Moreover, the rice variety Qingliu has been previously identified as an LF-resistant cultivar, which was found to have constitutive SA levels higher than the susceptible cultivar TN1 [41]. In light of our findings, SA signaling may be involved in rice resistance to LF by regulating downstream defense responses, such as rice peroxidase activity [40]. Another possibility is that SA itself acts as a defensive compound against LF, as this compound accumulates at high levels in rice, reaching up to 37 µg g^-1^ of fresh leaf weight [42]. Future experiments involving LF bioassays on rice SA signaling and biosynthesis mutants could be conducted to better understand the defensive role of SA against LF.

Flavonoids are widely distributed specialized metabolites in plants. While primarily known as medicinal agents and flavoring agents, some flavonoids play an important role in plant responses to abiotic and biotic stresses [32]. The role of flavonoids in herbivore resistance is increasingly being recognized. For example, in rice, BPH attacks result in a high accumulation of sakuranetin in the phloem of the leaf sheath. Disruption of the sakuranetin biosynthesis gene decreased plant resistance to BPH. Mechanistic studies revealed that sakuranetin can reduce the beneficial endosymbionts of BPH and in turn affect BPH performance [12]. In maize, a C-glycosyl flavone, maysin, was identified as a defensive compound against the lepidopteran maize earworm (*Helicoverpa zea*) [43]. In vitro artificial diet feeding assays reveal several other flavonoids with anti-herbivore activity [44, 45]. Naringenin is the core precursor of the most abundant flavonoids in rice. We found that P213 plants constitutively produce higher naringenin levels than XS11 plants. Many naringenin derivatives also robustly accumulate. We hypothesize that these compounds or their metabolites may have anti-LF activity. Unfortunately, the lack of a suitable artificial diet for LF larvae limits high-throughput screenings of anti-LF compounds. Therefore, future work should focus on identifying flavonoid biosynthetic genes and creating flavonoid-deficient mutants. Assessing LF performance on these mutants will help to determine the defensive role of these flavonoids.

In summary, this study examined the differences in chemical and physical defensive traits between an LF-resistant cultivar (P213) and a susceptible cultivar (XS11). JA-mediated defenses were inversely correlated with LF resistance in P213. On the other hand, SA levels, flavonoids, and silicified trichomes were all positively correlated with LF resistance. These findings expand our understanding of rice’s resistance against this insect pest and establish the foundation for mapping the genetic determinants of LF resistance.

## Materials and methods

### Plant materials and growth conditions

The japonica rice cultivars Xiushui 11 (XS11) and P213 were used. Seeds were germinated in plastic Petri dishes containing water and placed in an illuminated incubator at 28 ± 1°C with a photoperiod of 14 h L: 10 h D. After 7 days, the seedlings were transferred to a hydroponic solution, as previously described [46]. Plants were cultivated in a growth chamber under a 14 h light (28°C) and 10 h dark (26°C) photoperiod with 40-50% humidity. Four-week-old plants were used for experiments.

### Insect rearing

The colony of *Cnaphalocrocis medinalis* (rice leaffolder, LF) was initially obtained from a paddy field at Changxing agricultural experiment station of Zhejiang University (Huzhou, China). The LF population was maintained in a climate chamber at 25.5 ± 1°C with 14 h light and 65 ± 10% relative humidity. Larvae of similar size were used for experiments.

### LF bioassays

For the LF larval growth assay, freshly hatched larvae were allowed to feed on individual plants. Larval mass was recorded on days 11 and 15. Thirty biological replicates for each rice cultivar were used. For larval behavior assays, fourth-instar larvae with similar sizes and activities were selected and starved for 2 h before experiments. One larva was placed on each plant's first fully expanded leaf, and the time for starting to roll the leaf and spinning the first set of silks was recorded. The total number of silks spun per hour was counted. Fourteen biological replicates were used for each rice cultivar. For larval feeding assays, a third-instar larva starved for 2 h was allowed to feed on the first extended leaf for 24 h. Leaves were then excised and photographed, and the consumed leaf area was measured using ImageJ. Seven biological replicates were used for each rice cultivar. For the larval survival assay, two freshly hatched larvae were allowed to feed on a fully expanded leaf, and the number of surviving larvae on each plant was recorded after 8 d. Five plants (10 larvae) were set as one biological replicate, and five replicates for each rice cultivar were used.

### Phytohormone analyses

Leaf samples were ground into powder in liquid nitrogen, and approximately 100 mg of powder was (precise mass was recorded) extracted with 1 mL of ethyl acetate containing the internal standard (20 ng D6-JA and 5 ng D6-JA-Ile) as described previously [46]. Extracts were analyzed using LCMS-8040 (Shimadzu). JA and OH-JA were quantified using the internal standard D6-JA, and JA-Ile and OH-JA-Ile with D6-JA-Ile.

### Defensive compound measurements

For trypsin proteinase inhibitor (TrypPI) analysis, leaf materials were ground in liquid nitrogen, and approximately 50 mg of powder was homogenized with 300 μL of cold protein extraction buffer (0.1 M Tris-HC1, pH 7.6, 5% polyethylene polypyridine alkyl ketone, 2 mg/mL phenylthiourea, 5 mg/mL diethyldithiocarbamate, 0.05 M Na_2_EDTA) as described previously [27]. TrypPI activity was quantified by the radial diffusion method [47]. Six biological replicates were used in each treatment.

For phenolamide and flavonoid analysis, approximately 50 mg of leaf powder (mass was recorded precisely) was extracted twice with 800 μL of 70% methanol and 500 μL of 70% methanol, respectively. The supernatants of the two extracts were combined, and the organic phase was evaporated using an Eppendorf concentrator. The remaining aqueous phase was freeze-dried using a vacuum freeze dryer. The completely dried sample was dissolved in 150 μL of 70% methanol and analyzed by tandem LC‒MS using an electrospray ionization source (Agilent 6460). All the standards used in the experiments were of chromatographic grade and were obtained from Hangzhou Chemipanda Bio-Tech Co., Ltd. (China). The standard curve method was used to quantify each compound.

### Trichome analysis

Leaf samples were prepared and observed under a scanning electron microscope (SEM) to quantify the number and types of trichomes present. Leaf pieces of approximately 1 cm^2^ were carefully cut from the first fully expanded leaf of a 30-d-old rice plant. The samples were fixed with 2.5% glutaraldehyde solution at 4°C and 1% osmic acid. After fixation, the samples were washed three times (15 min per wash) with phosphate buffer (0.1 M, pH 7.0). Then, the samples were dehydrated using a graded ethanol series (30%, 50%, 70%, 80%, 90%, 95%, and 100%) with each concentration of ethanol for 15 min. The treated samples were dried in a Hitachi HCP-2 critical point dryer, and gold foils were sputtered on the surface of the samples using a Hitachi E-1010. Finally, the trichomes were observed and recorded under a Hitachi SU8010 scanning electron microscope. Four biological replicates for each rice cultivar were used.

For trichome observation in larval frass, the frass of fourth-instar larvae feeding on P213 was collected and crushed into small pieces using forceps. Trichomes were observed under a Keyence (VHX-7000) digital microscope.

### Silicon measurements

This experiment utilized microspectrophotometry to measure the silicon content in plant leaves. The first and second fully expanded leaves of each plant were collected as one biological replicate. Samples were ground to powder in liquid nitrogen and dried in an oven at 80°C. The silicon levels in each sample were analyzed by a plant silicon content assay kit (Suzhou Comin Biotechnology) according to the manufacturer’s instructions. The extracts were transferred into a transparent 96-well plate, and the absorbance at 650 nm was measured. Eleven biological replicates for each rice cultivar were used.

### Cellulose measurements

The first fully expanded leaf of each plant was collected. Samples were ground to powder in liquid nitrogen and dried completely in an oven at 80°C. Approximately 0.01 g of sample (precise mass was recorded) was dissolved in 1 mL of 80% ethanol solution and incubated in a water bath at 90°C for 20 min. The precipitates were washed with 1.5 mL of 80% ethanol and 100% acetone. The cellulose levels in the dried precipitates were analyzed by a cellulose content assay kit (Suzhou Comin Biotechnology) according to the manufacturer’s instructions. The extracts were transferred into a transparent 96-well plate, and the absorbance at 620 nm was measured. Ten biological replicates for each rice cultivar were used.

### Lignin measurements

The first fully expanded leaf of each plant was collected, and the sample was ground to powder in liquid nitrogen. Approximately 100 mg of the samples (exact mass was recorded) was extracted with 1 mL of methanol and incubated at 80°C for 2 h. The pellet was collected by centrifugation, washed with 1 mL of distilled water and resuspended in 750 μL of distilled water, 250 μL of concentrated HCl and 100 μL of thioglycolic acid. The mixture was incubated at 80°C for 3 h. After centrifugation, the pellet was washed and resuspended in 1 mL of 1 M NaOH at room temperature for 12 h. After spinning for 10 min at maximum speed in a microcentrifuge, the supernatants were transferred to a new tube, and 200 μL of concentrated HCl was added at 4 ℃ for 4 h to precipitate the lignin thioglycolic acid. The precipitates were collected by centrifugation and dissolved in 1 ml of 1 M NaOH, and the absorbance at 280 nm was measured. Six biological replicates for each rice cultivar were used.

### Transcriptome analysis

Total RNA was isolated using the MiniBEST Plant RNA Extraction Kit (TaKaRa). RNA sequencing was performed on an Illumina HiSeq platform by Novogene (https://www.novogene.com/). The low-quality reads of Illumina sequencing data and the adaptor sequences were filtered by TRIMMOMATIC l [48]. Then, the filtered reads were aligned to the rice reference genome using HISAT2 (http://rice.plantbiology.msu.edu/pub/data/Eukaryotic_Projects/o_sativa/annotation_dbs/pseudomolecules/) [49]. The read counts were subsequently normalized using STRINGTIE to obtain the TPM values (transcripts per million reads) [50]. Subsequently, the TPM values of all genes were subjected to principal component analysis (PCA) using the R package GGORD. The differentially expressed genes (DEGs) were analyzed using the R package edgeR (v.3.38.4) [51]. Gene Ontology (GO) enrichment analysis was performed using CLUEGO [52].

### Data analysis

The data obtained from the experiments were statistically analyzed using DPS software (http://www.dpsw.cn/dps_eng/). Student’s *t* test was used to compare the differences between two groups of samples, and the Duncan test in one-way ANOVA was used to compare the differences between multiple groups of samples. The asterisks (*) in the graphs indicate significant differences between groups (*, *p* < 0.05; **, *p* < 0.01; Student's *t* test); the groups marked with different letters in the graphs are significantly different from each other (*p* < 0.05, Duncan's multiple range test).

## Supplementary Information

**Additional file 1.**

## Data Availability

The RNA-seq data reported in this paper have been deposited in the Genome Sequence Archive at the BIG Data Center (http://bigd.big.ac.cn/gsa), Beijing Institute of Genomics (BIG), Chinese Academy of Sciences, under accession number CRA012291.
