# Supplementary material for: Enhanced chemical and physical defense traits in a rice cultivar showing resistance to leaffolder infestation
Source: Crop Health. 2023 Oct 23;1(1):10. doi: 10.1007/s44297-023-00010-z (PMC12825974; doi:10.1007/s44297-023-00010-z)
Supplement: Supplementary file 1 — Additional file 1. [file 44297_2023_10_MOESM1_ESM.docx]

**Supplementary Information**


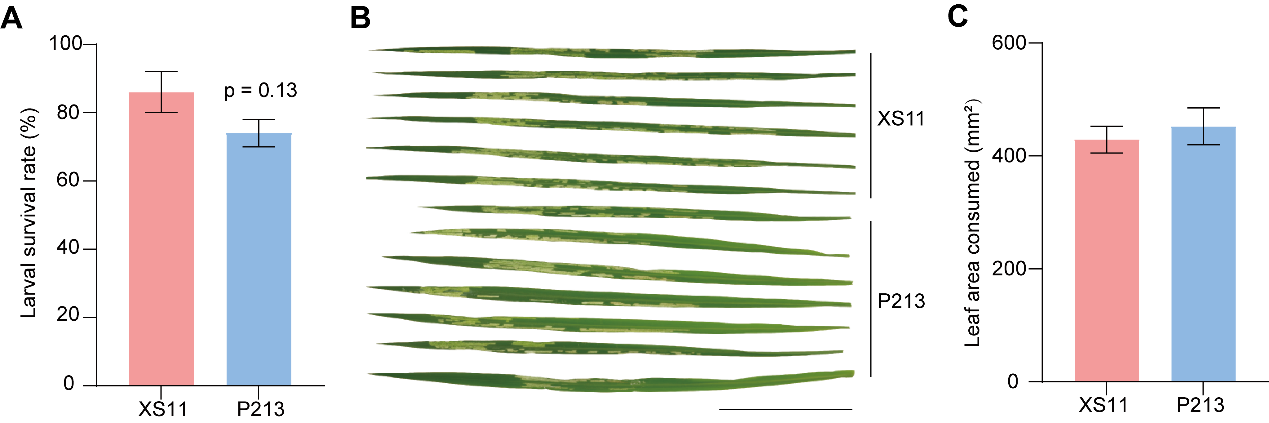


**Fig. S1** The leaf area consumed by fourth-instar LF larvae and the survival rate of freshly hatched LF larvae on P213 and XS11 plants. **A** The survival rate (± SE, n = 5) of fresh-hatched LF larvae fed on XS11 and P213 plants. **B** The damage of XS11 and P213 leaves fed by fourth-instar LF larvae for 24 h. Bar = 10 cm. **C** Mean leaf area (± SE, n = 6) consumed by LF larvae. Analysis was conducted by ImageJ software.


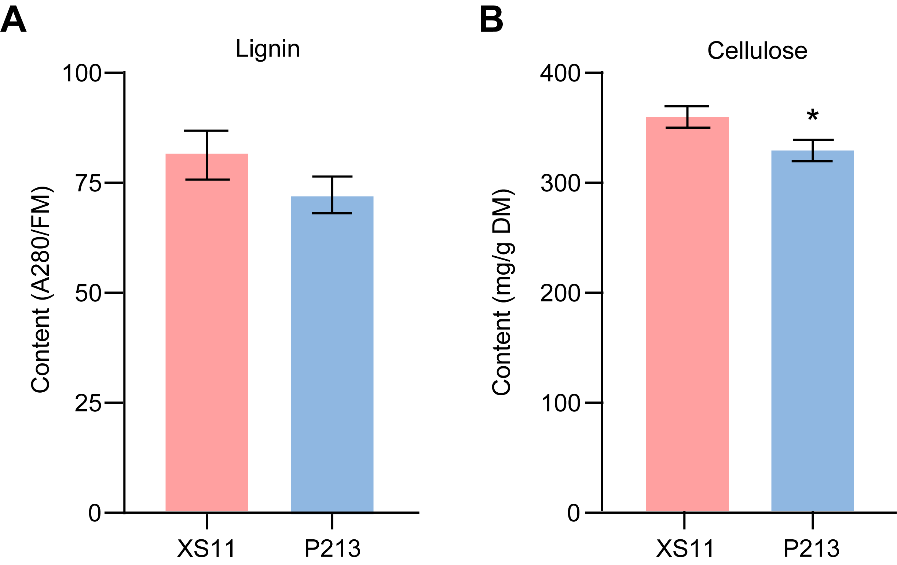


**Fig. S2** Lignin and cellulose content in the leaf of XS11 and P213 plants. Mean levels (± SE, n = 10) of lignin (**A**) and cellulose (**B**) in XS11 and P213 plants. Asterisks indicate significant differences in P213 compared with XS11 plants (*, P < 0.05; Student’s *t*-test). FM, fresh mass. DM, dry mass.

**Table S1. The TPM values of genes from RNA-seq data**

| **Gene ID** | **Name** | **XS11_0h** | **P213_0h** | **XS11_8h** | **P213_8h** |
| --- | --- | --- | --- | --- | --- |
| LOC_Os05g07090 | ACX | 3.8007912 | 2.6687195 | 6.1649056 | 4.7348595 |
| LOC_Os06g24704 | ACX | 4.2005699 | 3.3945768 | 6.5085393 | 4.8957045 |
| LOC_Os03g12500 | AOS2 | 1.0206084 | 0.1993238 | 2.295351 | 0.2707736 |
| LOC_Os08g39840 | HI-LOX | 1.4077573 | 0.7660686 | 5.2706388 | 3.0638327 |
| LOC_Os03g08320 | JAZ11 | 0.6880051 | 0.6337972 | 7.9273863 | 5.8567496 |
| LOC_Os09g26780 | JAZ8 | 1.3331355 | 1.7718414 | 8.7288401 | 7.2542146 |
| LOC_Os10g31950 | KAT | 4.0604177 | 3.3431283 | 9.011949 | 6.6808601 |
| LOC_Os12g37260 | LOX | 5.1369673 | 5.2163466 | 13.070336 | 9.7560889 |
| LOC_Os03g08220 | LOX | 5.0903716 | 4.4704453 | 6.6584095 | 5.290167 |
| LOC_Os08g39850 | LOX | 0.2349194 | 0.3478511 | 3.4402623 | 1.50211 |
| LOC_Os03g52860 | LOX | 0 | 0 | 6.6360405 | 0.5412261 |
| LOC_Os12g37350 | LOX | 2.1044042 | 2.5079726 | 9.574953 | 6.7473665 |
| LOC_Os02g17390 | MFP | 4.7328771 | 4.1608614 | 7.8518468 | 5.9910095 |
| LOC_Os06g11210 | OPR | 0 | 0 | 2.6067294 | 0.8743438 |
| LOC_Os05g50890 | JAR1 | 5.1403397 | 4.8256294 | 7.3138015 | 6.0249968 |
| LOC_Os06g08610 | PHT2 | 0.6680155 | 0 | 1.9388172 | 0.2554129 |
| LOC_Os09g37200 | PHT4 | 0.1157332 | 0 | 2.9199245 | 1.2020501 |
| LOC_Os12g27220 | SHT1 | 0.067943 | 0 | 8.6805935 | 0 |
| LOC_Os12g27254 | SHT2 | 0 | 0 | 6.9652432 | 0.7061534 |
| LOC_Os10g23310 | THT1 | 0 | 0 | 4.8355699 | 2.4251351 |
| LOC_Os01g03320 | PI | 5.4593641 | 3.5206721 | 12.55344 | 11.152369 |
| LOC_Os01g03680 | PI | 0 | 0 | 10.044447 | 7.5523451 |
| LOC_Os10g20890 | PI | 1.0999819 | 0.4136489 | 1.8361159 | 0.1497605 |
| LOC_Os06g49190 | PI | 1.949056 | 1.1994419 | 6.52541 | 4.689903 |
| LOC_Os03g57970 | PI | 0.2315086 | 0.1524957 | 3.530269 | 0.8320077 |
| LOC_Os07g09970 | PI | 5.4973858 | 4.045761 | 7.1181275 | 5.7552041 |
| LOC_Os02g37690 | UGT | 5.5789782 | 6.2311446 | 6.5045119 | 6.8191768 |
| LOC_Os06g09240 | UGT | 0.6182956 | 2.6300456 | 1.1238093 | 3.3223921 |
| LOC_Os05g45200 | UGT706E2 | 2.5603499 | 3.3806775 | 0.2456229 | 1.2330722 |
| LOC_Os01g53390 | UGT88C2 | 1.7162328 | 3.6694728 | 3.5796463 | 5.5124867 |
| LOC_Os02g41630 | PAL1 | 5.0607135 | 6.1690198 | 7.119342 | 6.86453 |
| LOC_Os02g41650 | PAL2 | 4.3074036 | 5.5012352 | 7.1315945 | 6.2356916 |
| LOC_Os02g41680 | PAL4 | 1.0751231 | 2.9026939 | 5.5770101 | 3.05058 |
| LOC_Os04g43800 | PAL6 | 0.1046266 | 1.0547247 | 6.3731088 | 5.2369627 |
| LOC_Os05g35290 | PAL7 | 1.012089 | 1.985889 | 5.6431288 | 3.2497592 |
| LOC_Os11g32650 | CHS1 | 1.7022903 | 4.1598015 | 3.4322426 | 5.4325415 |
| LOC_Os06g01250 | F2H | 3.0897355 | 4.0567583 | 5.8132034 | 6.9231217 |
| LOC_Os08g14760 | 4CL1 | 1.0668843 | 2.0545685 | 1.001423 | 2.3291549 |
| LOC_Os02g46970 | 4CL2 | 2.5727249 | 3.6260917 | 2.9768318 | 4.5021027 |
| LOC_Os02g28340 | MaT | 3.5496495 | 4.5326757 | 3.6693327 | 4.0931256 |
